# Supplementary material for: Self-sampling of capillary blood for SARS-CoV-2 serology
Source: Sci Rep. 2021 Apr 8;11:7754. doi: 10.1038/s41598-021-86008-5 (PMC8032656; doi:10.1038/s41598-021-86008-5)
Supplement: Supplementary file 1 — Supplementary Information [file 41598_2021_86008_MOESM1_ESM.docx]

**Self-sampling of capillary blood for SARS-CoV-2 serology: supplementary material**

Lottie Brown^1^, Rachel L Byrne^1^, Alice Fraser^1^, Sophie I Owen^1^, Ana I Cubas-Atienzar^1^, Chris Williams^1^, Grant A Kay^1^, Luis E Cuevas^1^, Joseph R A Fitchett^2^, Tom Fletcher^1,3^, Gala Garrod^1^, Konstantina Kontogianni^1^, Sanjeev Krishna^4^, Stefanie Menzies^1^, Tim Planche^4^, Chris Sainter^2^, Henry M Staines^4^, Lance Turtle^3,5^, Emily R Adams^1^*

1. Centre for Drugs and Diagnostics, Liverpool School of Tropical Medicine, Liverpool, L3 5QA, UK
2. Mologic COVID-19 Diagnostics Development Team, Thurleigh, Bedfordshire, UK
3. Tropical & Infectious Diseases Unit, Liverpool University Hospitals NHS Foundation Trust (member of Liverpool Health Partners), Liverpool, UK
4. Centre for Diagnostics and Antimicrobial Resistance, Institute for Infection & Immunity, St George’s University of London, London, UK
5. Dept of Clinical Infection, Microbiology and Immunology, University of Liverpool, Liverpool, L69 7BE, UK

Corresponding author: [emily.adams@lstmed.ac.uk](mailto:emily.adams@lstmed.ac.uk)

**Supplementary 1: Spearman’s rank coefficient and Bland-Altman Bias mean difference for all sample types positive for SARS-CoV-2 antibodies.**

| **Sample type (positives only)** | **N** | **Spearman r (95% CI)** | **P value** | **Bland-Atman Bias (SD)** |
| --- | --- | --- | --- | --- |
| Day 1 fridge | 18 | 0.8652 (0.6494 – 0.9520) | <0.0001 | 0.05963 (0.06961) |
| Day 1 RT | 17 | 0.8235 (0.5427 – 0.9387) | 0.0002 | 0.04084 (0.07189) |
| Day 3 RT | 16 | 0.7286 (0.3303 – 0.9066) | 0.0029 | -0.1114 (0.2150) |
| Day 5 RT | 16 | 0.6794 (0.2621 – 0.8827) | 0.0048 | -0.1411 (0.2281) |
| Day 7 RT | 16 | 0.7206 (0.3357 – 0.8993) | 0.0023 | -0.1881 (0.2431) |
| DBS | 15 | 0.5893 (0.09378 – 0.8508) | 0.0232 | 0.03457 (0.1761) |
| Total capillary | 95 | 0.6512 (0.5131 to 0.7564) | 0.0001 | -0.04734 (0.3191) |

**Supplementary 2: Bland-Altman mean difference comparison of OD ELISA results for matched capillary and venous blood samples (collected in lithium heparin treated tubes) (dashed lines represent 95% limits of agreement**

**(-0.6729 to 0.5782)**
